# Advanced practice and clinical supervision: An exploration of perceived facilitators and barriers in practice

**DOI:** 10.1111/jocn.16341

**Published:** 2022-04-27

**Authors:** Geraldine A. Lee, Edward E. Baker, Carolyne Stewart, Mary Raleigh

**Affiliations:** ^1^ Florence Nightingale Faculty of Nursing, Midwifery and Palliative Care King’s College London London UK

**Keywords:** advanced practitioners, barriers, clinical supervision, facilitators, supervision frameworks

## Abstract

**Aim and Objectives:**

The aim of this study was to investigate current advanced practice Masters students’ experience of clinical supervision, to explore how clinical supervision works in practice and to identify students’ perceptions of the facilitators and barriers to clinical supervision in their workplace.

**Background:**

Advanced practitioners, and in particular nurses, play a pivotal role in delivering health care across acute and primary care settings. These non‐medical professionals fulfil a rapidly expanding proportion of roles traditionally undertaken by medically qualified staff within the National Health Service in the United Kingdom and often lead specialist clinics and services. To prepare for the advanced practice role, individuals are required to undertake a Master's in advanced practice to develop the required skills and knowledge and work in clinical practice with a clinical assessor/supervisor to demonstrate competence and performance.

**Design:**

A mixed method study using an online descriptive cross‐sectional survey and qualitative data were collected via focus groups and has been reported using the Good Reporting of a Mixed Methods Study checklist.

**Results:**

A total of 79 students completed the online survey (from 145 AP students), a response rate of 55%. Most respondents were nurses (*n* = 73) with 49 (62%) in a formal advanced practice trainee role, and the majority believed their clinical supervisor had a good understanding of advanced practice and the advanced practice role. Two focus groups were held with 16 participants in total. Thematic analysis revealed five themes: (a) perceived level and amount of support from clinical supervisors, (b) skill level of clinical supervisors, (c) physicians and their perceptions on supervising, Advanced practitioners (d) clinical supervisors’ preparation for the role and (e) transition from trainee to qualified advanced practitioner.

**Conclusion:**

The survey revealed that advanced practitioner students perceived that clinical supervisors and workplace colleagues had a good understanding of the advanced practice role with good levels of support in practice. A more coherent approach is required for clinical supervision and an implementation framework that can be formally evaluated.

**Relevance to clinical practice:**

Several significant barriers to clinical supervision for advanced practitioner students were identified, and there are currently more barriers (including COVID‐19) than facilitators.

**What does this paper contribute to the wider global clinical community?**

- There is a need for a standardised supervision framework for APs in clinical practice.
- More work is required by employing organisations to recognise the need for protected time for clinical supervision for Aps.
- Formal clinical internship for APs would ensure protected time with a clinical supervisor and offers a structured approach to assessment of learning needs in clinical practice.
- Improved partnership working between healthcare providers and higher education institutions could support formal evaluations of supervision frameworks.
- It will take time for a body of APs to be trained as supervisors, so in the meantime supervisor, training for medical practitioners is recommended as part of their continuous professional development.

## 1 INTRODUCTION

Advanced level practitioners have been part of the United Kingdom nursing workforce since the 1980s. Over time, the role has expanded and there is now robust evidence of the effectiveness of the role from a variety of settings and specialties (Moxham and McMahon‐Parkes, 2000). The percentage of nursing vacancies in 2019 has risen from 7.2% to 9.4%, demonstrating the increased number of nursing and midwifery posts required to meet demand (NHS Digital, n.d.).

A 2016 report on reshaping the workforce highlighted the need to train, recruit and upskill the workforce to ensure the NHS evolves to meet the health needs of the population (Imison et al., 2015), which has resulted in the advanced practice role developing rapidly in recent years. In 2017, Health Education England (HEE) developed a new definition for advanced practice (AP) describing its scope, standardising the role functions, educational preparation, practice capabilities and role development. The four pillars that underpin this practice are 1. Clinical Practice 2. Leadership and Management 3. Education and 4. Research. HEE states that.

*‘Advanced Clinical Practice is delivered by experienced registered healthcare practitioners. It is a level of practice characterised by a high level of autonomy and complex decision‐making. This is underpinned by a Masters level award or equivalent that encompasses the pillars of clinical practice, management and leadership, education and research, with demonstration of core and area specific clinical competence’*. (7)

This definition has now been adopted across England and Wales to standardise the role and allow consistent practice across disciplines for AP roles (HEE, 2017) with the aim to meet HEE’s objective to develop a flexible workforce, which is competent to respond to changing healthcare needs. Allied health professionals (such as paramedics and physiotherapists) are now employed in AP roles to meet the need for more healthcare professionals to undertake diagnostic and decision‐making roles, traditionally performed by physicians (Health Education England (HEE), 2017; Imison et al., 2015). AP roles offer opportunities to not only address shortages within the medical workforce, but also to improve clinical continuity and to provide new opportunities for non‐medical healthcare professionals through new ways of working in clinical practice.

The AP framework aimed to standardise the advanced practitioner role with a clear definition and core capabilities. All registered healthcare professionals should be continuously developing their practice, and so it is anticipated that APs working at advanced level will develop their practice beyond this threshold by working with a suitable clinical supervisor in their workplace.

The role of the clinical supervisor is to provide opportunities for the AP to reflect on and review their clinical practice, discuss individual cases in depth and identify changes or modifications to practice required to maintain professional and public safety. Clinical supervision also provides an opportunity to identify training and continuing development needs (Care Quality Commission (CQC, 2013)). The Royal College of Nursing debated that a fellow registered nurse could undertake the role as a clinical supervisor for the AP, but this view was not supported because the developing AP is more advantaged by working with, and alongside a range of other multi‐professionals who have a broader range of skills and higher levels of theoretical knowledge (RCN, 2019).

The expectation is that APs working at advanced level will have achieved this during extensive experience and following completion of master's level education or its equivalent. As such, AP students require clinical supervisors to assess them in their workplace and ensure they are competent in all the core capabilities. Competence is a concept around the AP’s knowledge, skills, values and attitudes (Raleigh & Allan, 2017). A recently released report noted that there is wide variation in the establishment of APs and current supervision practices are usually profession‐specific with some supervision across the traditional professional boundaries (HEE, 2020). This report also proposes that clinical supervision is perhaps best led by the trainee AP because their learning needs are individualised, and they may require different supervisors at different points in their development journey (HEE, 2020). Nevertheless, a structure to guide the supervision process has been proposed (HEE, 2020), whereby trainee APs should identify a suitable assessor within their work environment. The assessor should.

- Have a good understanding of the advanced practice role
- Is up‐to‐date and have clinical expertise in your specialty
- Available during clinical placement and commit to being a supervisor with a commitment to meeting once a week
- Able to observe the student working clinically and provide thorough critical feedback on their performance in the role
- Accessible within the clinical environment for teaching and supervision and reviewing patients seen by the student

## 2 BACKGROUND

Most of the clinical supervision literature on nursing has focused on under‐graduates with a paucity of studies for advanced practice. Many of the papers on AP focused on the transition from trainee to qualified practitioner (Dover et al., 2019; Murphy & Mortimore, 2020). A recent study in primary care applied a ‘hub and spoke’ approach as an alternative to traditional supervision methods in primary care. This process used the strengths of both the General Practitioner (GP) and the AP (Gloster et al., 2020). The ‘hub and spoke’ primary care model is an organisation where a main well‐resourced hub is established that supplies intensive medical services and is complemented by satellite campuses/spokes. In the findings of their qualitative analysis, it was noted that trainee ACPs highly valued consistent clinical support from a range of designated GP supervisors and peers afforded by the ‘hub and spoke’ model, because they worked across different practice boundaries (Gloster et al., 2020). Trainee ACPs experienced better levels of clinical supervision when GP supervisors understood supervision requirements, and the vision of the trainee ACP role in primary care (Gloster et al., 2020).

One Australian paper examining clinical supervision in allied health professionals reported survey data on 153 respondents highlighting the value of clinical supervision. The study noted barriers with a lack of time, variable understanding of clinical supervision, limited access to supervisors, efficacy of clinical supervision and policy implementation (Wilson & Taylor, 2019). A focus group of 23 clinicians was also performed, and thematic analysis revealed 5 themes: the importance of clinical supervision, variable understanding of clinical supervision, limited or no participation in clinical supervision, perceived lack of efficacy with supervision received and limited awareness of supervision policy.

The AP NHS England Framework introduced capabilities as part of the AP Masters programmes and as such, students must be assessed in their clinical workplace and deemed competent in all areas (HEE, 2017). In our institution, students are required to identify a suitable clinical supervisor who is willing to support and assess them in practice using the AP framework document and using the King's Performance Rating Criteria, so the clinical supervisor can document the level students are working at (Fitzpatrick et al., 1997). This scale allows students and supervisors to identify the areas of independent practice and those of a supervised, assisted and dependent level of practice. The amount and level of clinical supervision have not been quantified, and there is no information available on the barriers and facilitators to clinical supervision or the perceptions of AP students on clinical supervision.

The supervisor role is either a consultant physician or a senior clinical nurse (nurse consultant or advanced practitioner, for example as laid out in the university ACP documents and following the HEE supervisory document). We were seeking to determine whether there was informal feedback sought by students and their formal feedback as required by the clinical competency document which must be submitted at the end of each year.

## 3 METHODS

The aim of this study was to investigate current AP students’ experience of clinical supervision in their workplace, explore how clinical supervision works and identify students’ perceptions on the facilitators and barriers to clinical supervision. These will be explored through:

- The views and perceptions about clinical supervision from current AP students
- The identification of facilitators and barriers relating to clinical supervision for AP students

### 3.1 Ethics considerations

This project has received ethical approval by the Kings College London Research Ethics Committee (REC) Reference: MRA‐19/20‐17189.

### 3.2 Study design

A mixed method approach was used to examine AP students’ perception with quantitative data collected via an online descriptive cross‐sectional survey and qualitative data collected via focus groups. Study findings have been reported through the Good Reporting of a Mixed Methods Study (GRAMMS) checklist (O’Caithain et al., 2008. Supplementary File 1).

### 3.3 Setting participant selection

Inclusion criteria: Students currently enrolled on the MSc AP programme at Kings College London, and the exclusion criteria were any student not enrolled in the AP programme. The survey was open to all students enrolled (approximately 145) who were in their first or second year of their studies. With regard to the focus groups, the aim was to hold one to three focus groups, each with approximately 6–10 students.

### 3.4 Participant recruitment

An announcement was distributed to all currently enrolled AP students via the university AP virtual learning platform. A study information sheet was attached and those interested emailed the Principal Investigator to participate in the focus group. Written informed consent was gained prior to each focus group. For the survey component, a link was supplied in the announcement and consent for the survey was assumed if students completed it online using Microsoft Polls. Participants were made aware that it would not be possible to withdraw data submitted through the online survey portal after submission due to the anonymous submission process. Participants attending a focus group session were informed that it would not be possible to withdraw their recorded data after recording of the session had started, but the participants right to refrain from answering (a) question(s) or leave the focus group event were reiterated to all participants prior to the focus group starting.

### 3.5 Survey measures

As no previously tested questionnaire was available, the survey was developed through a process of literature review, discussion with members of the target population and members of the author team (Kelley et al., 2003). The survey was developed using best practices and a group consensus on content was gained from all authors. The questionnaire was piloted by the main author with a small number of academics in the Faculty and three alumni who had completed an advanced practice Masters, and feedback was integrated through questionnaire revisions. The final survey contained 5 questions relating to demographics and 11 questions relating to their experience of supervision in clinical practice. Although this was primarily a quantitative section, participants were encouraged to make comments about the answers they had provided.

### 3.6 Focus groups

Focus group discussions were conducted by two members of the research team as per best practice (Jamieson & Williams, 2003). One researcher moderated the group discussion, while the second acted as a non‐participant observer and note‐taker. Data collected at the focus groups were recorded and transcribed. A topic guide was used to encourage participants to reflect on their own experience of clinical supervision and share their views and critically explore clinical supervision. Table 1 presents the topic guide used in the study.

**TABLE 1:** Focus group question guide

Q1. In what capacity/ structure, do you work with your clinical supervisor?
Q2. In an average week, how much time would you spend working with your clinical supervision?
Q3. Tell us about how you work with your clinical supervision?
Q4. Have there been any issues and if so, how have they been resolved?
Q5. What do you think about having just one clinical supervision?
Q6. Do you seek feedback from other colleagues? If so, is it formal or informal?
Q7. Would case‐based scenario discussions be useful in practice as part of your clinical supervision?
Q8. If you could implement or change one aspect of clinical supervision, what would it be?
Q9. Do you think it is important to have clinical supervision as part of the AP?
Q10. Do you think a formal clinical supervision course would be useful for your supervisor & their understanding of AP?
Q 11. Are there any other comments, you would like to make on clinical supervision?

### 3.7 Data analysis

Quantitative data analysis was undertaken on survey data through the survey platform. Descriptive statistics were used to describe the participant characteristics and their experience of supervision through counts and percentages.

Management of the qualitative data involved interim analysis at the end of each focus group to highlight emerging themes and determine data saturation. Following transcription, audio‐recorded data were amalgamated with written field notes. Qualitative data were imported into NVivo (QSR International, 2020) and subjected to a standard process of inductive thematic content analysis (Braun & Clarke, 2013). Final categories and themes were determined using a consensus approach by the authors’ team to resolve any differences in interpretation. Anonymised quotes, which highlight key issues of interest, were reported as part of the results. Final themes were agreed using a consensus approach by all members of the project team.

Mixed method integration of data was undertaken using a triangulation design and a convergence model. This involved initial analysis of qualitative and quantitative results in isolation. Following this, results were compared and similarities or contrasting findings were considered together in the final analysis and discussion.

## 4 RESULTS

### 4.1 Survey

A total of 79 students completed the online survey out of 145 AP students equating to a response rate of 55%.

Most respondents were nurses, and other participants were pharmacists (*n* = 5) and physiotherapist (*n* = 1) (see Table 2) with 49 in a formal AP trainee role. A total of (*n* = 42) were funded by Health Education England as each area is given £2500 per student per year for their clinical supervision.

**TABLE 2:** Survey responses about AP role and clinical supervision (*n* = 79)

What is your profession and specialty?	
Nurse	73
Pharmacist	5
Physiotherapist	1
Are you in a formal AP trainee role?	
Yes	49
No	30
Funding source	
Health Education England	42
Self‐funding	13
Other	21
Not answered	3
Do you get protected time as part of your role?	
Yes	45
No	31
Not answered	3
What profession is your supervisor?	
Nurse	24
Physician	52
Allied health professional	3
Do you seek feedback from other colleagues?	
Yes	78
No	1

A total of 45 respondents stated they had protected time for their studies, which they described as relating to their formal university study or to their clinical practice. A total 17 respondents reporting 7.5 hours per week in the comments section with others reporting 1.5 hours a week protected time. Protected time was time that was allocated for their studies, which could relate to their formal university study or relating to their clinical practice. The remaining 27 students had a senior nurse practitioner/nurse consultant or senior clinical healthcare professional (i.e. Physiotherapist) as their supervisors.

Two‐thirds (*n* = 52, 66%) had a medical physician as their clinical supervisor, and all bar one respondent sought feedback from their clinical colleagues (i.e. those in more senior positions such as medical consultants, GPs or Band 8 nurses or allied health professionals).

Respondents were asked about the experience and suitability of their clinical supervisor, and the majority reported that they thought their supervisor had a good understanding of AP and the AP role (*n* = 66, 84%) and were familiar with the ACP framework (*n* = 56, 71%) (See Table 3). Over half of respondents thought their workplace colleagues had a good understanding of the role (*n* = 44, 56%), and similar numbers believed their supervisor did not need to undertake any formal supervisory courses (*n* = 42, 53%). We asked respondents whether they believed that medical consultants placed the same level of importance on clinical supervision for APs as they do for junior doctor training with 37 responding negatively (47%).

**TABLE 3:** Survey responses on suitability and experience of supervisors

Do you think your supervisor has a good understanding of AP and AP role?	
Yes	66
No	6
Don't know	7
Is your supervisor familiar with the NHS England AP framework?	
Yes	56
No	10
Don't know	13
Do you think your workplace colleagues have a good understanding of the AP role?	
Yes	44
No	24
Don't know	11
Do you think your clinical supervisor needs to undertake a formal supervisor course?	
Yes	16
No	42
Don't know	14
They have completed a course	7
Do you think that medical consultants place the same level of importance on clinical supervision for APs as they do for junior doctor training?	
Yes	28
No	37
Don't know	1

Under the free text section, several respondents commented on the impact of the COVID‐19 pandemic on their ability to meet and work with their clinical supervisors:

‘*I had some difficulties in getting protected time with my supervisor during the peak of the pandemic of COVID‐19’*.

*‘It has been difficult to undertake certain tasks due to the pandemic ‐ however both my clinical supervisor and area of work have been supportive. They are able to accommodate my learning if well planned’*.

‘*During and post pandemic with redeployment and new ways of working, it has meant clinical supervision opportunity has lessened. Hope to have a structured supervision timetable set up’*.

Due to the COVID‐19 pandemic, we were unable to undertake further focus groups in March due to lockdown; however, we managed to undertake two focus groups and analyse data on 16 participants. Following coding and thematic analysis, several themes were evident from the focus groups:

#### 4.1.1 Interviews

Five major themes emerged pertaining to support from clinical supervisors: (a) perceived level and amount of support from clinical supervisors, (b) Skill level of clinical supervisors, (c) Student perceptions of Physicians supervising APs, (d) Clinical supervisors’ preparation for the role and (e) Transition from trainee to qualified AP.

#### 4.1.2 Perceived level and amount of support from clinical supervisors

Many reported a positive experience of clinical supervision which resulted in greater confidence and competence:

*‘I* *have been well supported in practice*. *I feel that this has improved my confidence as I can have feedback from different clinicians and work alongside GPs and ANPs’*.

The amount of time spent with clinical supervisors varied greatly amongst focus groups participants. Some reported informal structures in place, whereas others had more structured formal clinical supervision.

*‘…it’s just like after clinic sessions finish, if I’ve had a few problematic patients or cases and I’ll be taking my supervisors opinion of, was this the best, sort of, diagnosis, what do I need to do further to follow it up, what tests should I have ordered, but that’s more informal’*.

For some in General Practice, they consulted their supervisor as required and had protected time:

*‘… [be]cause I’m in general practice, I end up going and seeking advice after the clinical sessions … after you’ve seen the patient, assessing how I’ve done… but then I also get an hour’s protected time a week’*.

The one universal comment from participants was the need for more time with their clinical supervisors. The problem with time pressures was evident and some participants shared experiences of group supervision:

’*It is very difficult to arrange protected time as my supervisor would be allocated to an operating list on the days that she is on*‐*site*. *The demand in both our roles changed so many times due to clinical need… that supervision did not always go as planned*. *Not working in an AP role makes it very difficult to achieve outcomes*’.

#### 4.1.3 Skill level of clinical supervisors

The issue of who should be a clinical supervisor in terms of their appropriateness for the role was discussed in the focus groups. Some participants felt that there were no suitably qualified nurses who could act as their supervisor for AP:

*‘So, we’ve got other people in the same sort of situation, where you’re seeking someone who’s a senior nurse that just doesn’t have the academic or the clinical skills to be able to manage’*.

Whereas others have adequate support from nursing colleagues, particularly where advanced roles such as the consultant practitioner role were established:

*‘I* *have a lot of support from nurse consultants in the department’*.*’*

The participants felt strongly about who should be a clinical supervisor and agreed that physicians or qualified ACPs were the most appropriate as they also had the clinical specialty knowledge:

*‘So*, *I think actually get the medics involved cause actually we're going to be*, *you know*, *potentially their workforce’*.*’*

#### 4.1.4 Student perceptions of physicians supervising APs

The participants highlighted the conflicting demands of physicians in the acute clinical setting identifying this as a barrier to effective supervision and clinical learning:

*‘My supervisor is an anaesthetist and a consultant for HDU. She is very dedicated and because there is no protected time, we meet in our own time and the day she is working on the unit as the consultant, I shadow her if both our scheduled work allows’*.

*‘They're* *busy with training the doctors and the SHOs rather than focusing on the AP’*.

This barrier was less an issue for those who work in General Practice where there was often more time for protected supervision:

*‘In primary care, they are more likely to support you because there isn’t a hierarchy there, so that makes it easier to go to them for support, but scheduling time is still an issue’*.

However, for some AP students, their supervision was less than optimal, and the role not fully understood by their supervisors. For some participants, aligning the AP learning needs with that of medical students helped improve the supervisors understanding:

‘*I think that the doctors need to take the role more seriously and give us the same attention as they do their medical students. It would also be nice for proper one‐on‐one time set aside with our mentors as that never seems to happen’*.

#### 4.1.5 Clinical supervisors’ preparation for the role

The level of preparation of clinical supervisors was another theme. Some participants highlighted the need for the university to provide education and preparation sessions for prospective clinical supervisors:

*‘But the key thing was if it was a webinar or something, they could tap into and then access, which I think would also help maybe if there were kind of updates for supervisors of what they should be doing throughout the year not just a one off [training]’*

One participant raised the issue of supervisors completing formal supervision training prior to undertaking clinical supervision:

*‘Or maybe they shouldn’t be supervisors until they have done it [formal supervision training], how you’re not to mentor student nurses until you have done your proper mentoring [course]’*

*‘I* *definitely think the supervisors should have a course and updated regularly so that they will have a good understanding about AP role and the recent changes’*.

Suggestions were made on how clinical supervision could be improved using a more standardised session structure and standardised documentation approach:

*‘I think a standardized AP supervision template would be helpful. As it stands, local trusts design their own and often tailored to junior doctors. A standardized guide will enable supervisors know what it is they are to achieve during supervision’*.

‘*Great opportunity for learning and collaboration. More formalisation/structure would be beneficial for both the supervisor and the student’*.

#### 4.1.6 Transition from trainee to qualified AP

The issue of transitioning from an AP student to a fully qualified AP was raised: This relates to students’ gaining confidence during their MSc and as such being able to transition from a trainee and this is reflected in the student quotes:

*‘I* *think it would be good to have a transition*, *I think a transition would definitely be worthwhile’*.

One unexpected theme was the role of current AP students supporting other members of the workforce including junior doctors, demonstrating the increasing role modelling and leadership as they progress through the AP programme:

*‘Well, I think that’s the other thing is, especially with having the AP they now get you to support the registrars that are coming through as well so it’s not just you just with your own kind, it’s about you now supporting the wider workforce. So, I’m supporting not only pharmacists, I’m supporting registrars with their training and I’m supporting the nurses, the trainee healthcare assistants who are being upskilled so it’s supporting the wider workforce rather than just specifically your own and I think that’s the way healthcare is going’*.

## 5 DISCUSSION

This mixed method study was able to explore the experience of AP students and clinical supervision. The survey identified that half of respondents were in formal AP training roles and just half had protected time and 70% of AP trainees had physicians as their clinical supervisor. The survey respondents thought that their clinical supervisors were familiar with the AP framework and over 80% had a good understanding of the AP role. To the best of our knowledge, this is the first study examining the AP students’ perceptions of clinical supervision using the AP framework in the UK.

Overall, the perceptions of the AP students on their supervisors were positive. A recent paper reported physician leadership predicting AP job satisfaction in a US study of 320 advanced practitioners (Guevara et al., 2020). Physician transformational leadership was able to predict the variance of job satisfaction between 4.4 and 49.1%. Given our students reported that the majority had physicians as their supervisors, it suggests that developing them as leaders for AP is worthwhile until the number of qualified APs who can be clinical supervisors increases.

Five themes were identified through the focus groups: Perceived level and amount of support from clinical supervisors, skill level of clinical supervisors, advanced practitioners’ perceptions of physicians on supervising them, clinical supervisors’ preparation for the role and transition from trainee to qualified AP. Several of these themes have previously been reported in the literature and lack of time for clinical supervision is always a major concern (Dover et al., 2019; Murphy & Mortimore, 2020; Sharrock et al., 2013; Wilson & Taylor, 2019). Organisational approaches to improving clinical supervision have been suggested in reducing these barriers (Wilson & Taylor, 2019). Heavy workload and conflicting demands of both AP students and their supervisors are recognised barriers to supervision, but with the current vacancies in the NHS and the ongoing pandemic, it is difficult to see how these can be resolved in the near future. Nevertheless, exploring the supervisor's perspectives to know how they managed workload and supervision demands during the pandemic may shed some now insights as to how this can be managed in more innovative ways such as in a virtual capacity that includes virtual out‐patient clinics (telephones) and remote consultations. However, with regard to facilitators during this study, the AP students were positive about their supervisors in terms of their understanding of the role and this suggests that recognition of the role is gaining although this was not formally evaluated.

Educational preparedness for transition from AP trainee to AP has previously been recognised as a major barrier in the literature (Dover et al., 2019). The issue of clinical supervision for transition has highlighted the need for supportive relationships and the need for supervisors during this role transition (Sharrock et al., 2013). One paper suggests that and having a clinical supervisor, students should have a mentor who supports learning and development of competencies (Murphy & Mortimore, 2020). One solution is use of a clinical internship which ensures the protected time with a clinical supervisor and offers a structured approach to assessment of learning needs in clinical practice (Lee & Fitzgerald, 2008). A hub and spoke model has been proposed for primary care AP training, and the three themes identified were support, supervision and vision (Gloster et al., 2020). Another possibility is increasing the partnerships between higher education and health care (Haggman‐Laitila & Rekola, 2014) and undertaking further evaluation of clinical supervision models in advanced practice (Lee & Metcalf, 2009). A recent guide on clinical supervision has been released by HEE, and this will aid in the understanding of clinical supervision and what clinical supervisors need to do in practice.

## 6 CONCLUSION

The mixed method approach provided adequate new information on issues within the clinical workplace and identified some facilitators and barriers to clinical supervision of AP trainees. It is clear a more coherent approach is required for clinical supervision and an implementation framework that can be formally evaluated including supervision session structure and a standardised documentation tool, which could be directly transferable between different healthcare organisations and universities.

## 7 RELEVANCE TO CLINICAL PRACTICE

This study was conducted through one university and as such may not be representative of other higher education institutes. However, our students work in a variety of healthcare settings in a metropolitan area in both acute and primary care, and therefore, we believe they are representative of master's students enrolled in AP programmes. Due to the COVID‐19 pandemic, we were unable to undertake further focus groups due to lockdown; however, we managed to undertake 2 focus groups and analyse data on 16 participants.

## CONFLICT OF INTEREST

Nil.

## AUTHOR CONTRIBUTION

GL, EB, CS and MR made substantial contributions to conception and design, or acquisition of data, or analysis and interpretation of data; involved in drafting the manuscript or revising it critically for important intellectual content; given final approval of the version to be published. Each author should have participated sufficiently in the work to take public responsibility for appropriate portions of the content; and agreed to be accountable for all aspects of the work in ensuring that questions related to the accuracy or integrity of any part of the work are appropriately investigated and resolved.

## Supporting information

Supplementary Material

## Data Availability

The authors confirm that the data supporting the findings of this study are available within the article and its supplementary materials.
