# Supplementary material for: Advanced practice and clinical supervision: An exploration of perceived facilitators and barriers in practice
Source: J Clin Nurs. 2022 Apr 27;32(5-6):780–8. doi: 10.1111/jocn.16341 (PMC10084051; doi:10.1111/jocn.16341)
Supplement: Supplementary file 1 — Supplementary Material [file JOCN-32-780-s001.docx]

**The quality of mixed methods studies in health service research**

**(GRAMMS Checklist)**

(Available: <https://www.equator-network.org/reporting-guidelines/the-quality-of-mixed-methods-studies-in-health-services-research/>)

(Developed from: O'Cathain A, Murphy E, Nicholl J. The quality of mixed methods studies in health services research. J Health Serv Res Policy. 2008;13(2):92-98.)

|  | Yes/No |
| --- | --- |
| **Assessment of the success of mixed methods studies in Health Service Research** | |
| Is the quantitative Component feasible? | Yes |
| Is the qualitative component feasible? | Yes |
| Is the mixed methods design feasible? | Yes |
| Have both qualitative and quantitative components been completed? | Yes |
| Were some quantitative methods planned but not executed? | No |
| Were some qualitative methods planned but not executed? | Yes (Focus groups due to Covid) |
| Did the mixed methods design work in practice? | Yes |
| **Assessment of the Mixed Methods design in health services research** | |
| Is the use of mixed methods research justified? | Yes |
| Is the design for mixing methods described? | Yes |
| Is the design clearly communicated? | Yes |
| Is the design appropriate for addressing the research questions? | Yes |
| Has the rigour of the design been considered or adhered to? | Yes |
| **Assessment of the quantitative component of Mixed methods design in health services research** | |
| Is the role of each method clear? | Yes |
| Is each method described in sufficient detail? | Yes |
| Is each method appropriate for addressing the research question? | Yes |
| Is the approach to sampling and analysis appropriate for its purpose? | Yes |
| Is there expertise among applicants/authors? | Yes |
| Is there expertise on the team to undertake each method? | Yes |
| Have issues of validity been addressed for each method? | Yes |
| Has the rigour of any method been compromised? | No |
| Is each method sufficiently developed for its purpose? | Yes |
| Is the (intended) analysis sufficiently sophisticated? | Yes |
| **Assessment of the qualitative component of Mixed methods design in health service research** | |
| Is the role of each method clear? | Yes |
| Is each method described in sufficient detail? | Yes |
| Is each method appropriate for addressing the research question? | Yes |
| Is the approach to sampling and analysis appropriate for its purpose? | Yes |
| Is there expertise among the applicants/authors? | Yes |
| Is there expertise on the team to undertake each method? | Yes |
| Have issues of validity been addressed for each method? | Yes |
| Has the rigour of any method been compromised? | No |
| Is each method sufficiently developed for its purpose? | Yes |
| Is the (intended) analysis sufficiently sophisticated? | Yes |
| **Assessment of integration in mixed methods studies in health service** | |
| Is the type of integration stated? | Yes |
| Is the type of integration appropriate to the design? | Yes |
| Has enough time been allocated for integration? | Yes |
| Is the approach to integration detailed in terms of working together as a team? | Yes |
| Does the dissemination strategy detail how the mixed methods will be reported in final reports and peer-reviewed publications? | Yes |
| Are the personnel who participate in the integration clearly identified? | Yes |
| Did appropriate members of the team participate in integration? | Yes |
| Is there evidence of communication within the team? | Yes |
| Has rigour been compromised by the process of integration? | Yes |
| **Assessment of inferences made in completed reports of Mixed methods studies in health services research** | |
| Is there clarity about which results have emerged from which methods? | Yes |
| Are inferences appropriate? | Yes |
| Are the results of all the methods considered sufficiently in the interpretation? | Yes |
